# Overexpression of a DNA Methyltransferase Increases Persister Cell Formation in Acinetobacter baumannii

**DOI:** 10.1128/spectrum.02655-22

**Published:** 2022-11-23

**Authors:** Hyunkeun Kim, Jee Hong Kim, Hongbaek Cho, Kwan Soo Ko

**Affiliations:** a Department of Microbiology, Sungkyunkwan University School of Medicine, Suwon, Republic of Korea; b Department of Biological Sciences, Sungkyunkwan University, Suwon, Republic of Korea; Emory University

**Keywords:** persister, epigenetic, DNA methylation, *dam*, *recC*, *Acinetobacter baumannii*, *persister*

## Abstract

Many mechanisms have been proposed to be involved in the formation of bacterial persister cells. In this study, we investigated the impact of *dam* encoding DNA methylation on persister cell formation in Acinetobacter. We constructed plasmids overexpressing *dam* encoding DNA-(adenine N6)-methyltransferase and four genes as possibly involved in persistence and introduced them into three A. baumannii strains. For persister cell formation assays, bacteria were exposed to ciprofloxacin, imipenem, cefotaxime, and rifampin, and the transcription levels of the genes were measured by qRT-PCR. In addition, growth curves of strains were determined. We found that all five genes were upregulated following antibiotic exposure. Dam overexpression increased persister cell formation rates and activated the four persister cell-involved genes. Among the four persister cell-involved genes, only RecC overexpression increase persister cell formation rates. While *recC*-overexpressing strains showed higher growth rates, *dam*-overexpressing strains showed decreased growth rates. In this study, we revealed that a DNA methyltransferase may regulate persister cell formation in A. baumannii, while RecC seems to mediate epigenetic regulation of persister cell formation. However, Dam and RecC may act at different persister cell formation states.

**IMPORTANCE** Bacterial persister cells are not killed by high concentration of antibiotics, despite its antibiotic susceptibility. It has been known that they may cause antibiotic treatment failure and contribute to the evolution of antibiotic resistance. Although many mechanisms have been suggested and verified for persister cell formation, many remains to be uncovered. In this study, we report that DNA methyltransferase leads to an increase in persister cell formation, through transcriptional activation of several regulatory genes. Our results suggest that DNA methyltransferases could be target proteins to prevent formation of persister cells.

## INTRODUCTION

Bacterial persister cells were first observed by Hobby et al. (1942); they found that a small subpopulation (<1%) of Staphylococcus aureus cells survived killing by penicillin. A couple of years later, this novel bacterial phenomenon was named “bacterial persistence” by Joseph Bigger (1). Later, persister cells were reported in several bacterial pathogens, including Escherichia coli (2, 3), Mycobacterium spp. (4), Pseudomonas aeruginosa (5, 6), and Acinetobacter baumannii (7, 8). Persister cells are now defined as subpopulations of cells within growth-arrested bacterial cells that are extremely tolerant to a variety of harsh environmental pressure sources, including antibiotics (9). It has been repeatedly recognized that bacterial persister formation is clinically significant because persister cells may cause antibiotic treatment failure and may contribute to the evolution of antibiotic resistance. While bacterial persisters have been studied over the last decade (10), understanding the phenomenon and its mechanisms is still in the beginning stages.

It has been suggested that the process of persister cell formation is distinguished into two major stages: dormancy and resuscitation states (11). In the hypothesis, bacterial dormant cells are induced by extremely harsh environmental conditions, including antibiotic exposure, to which cells respond by minimizing their metabolism and stopping replication. Rather than a spontaneous process, resuscitation would be the response to an improvement in environmental conditions; it depends on ribosome activity (11). Single-cell observations demonstrated that persister cell waking involves nutrient sensing by the chemotaxis system and phosphotransferase membrane proteins in E. coli (12).

Many genes, pathways, and systems have been shown to be involved in the development of bacterial persister cells. Regarding the toxin-antitoxin (TA) system, *hipA7*, *mqsR*/*mqsA*, *tisB*/*istR-1*, and *yafQ*/*dinJ* genes have been reported to be associated with persister cell formation (13–16). The alarmone guanosine tetraphosphate (ppGpp), which is synthesized by RelA and SpoT, was also reported to be associated with the TA system and persistence (17, 18). PhoU, involved in phosphate metabolism, negatively regulates numerous genes involved in energy production, which is known to be correlated with persister cell formation in E. coli (19). In addition, energy metabolism and SOS responses have also been shown to be associated with persister cell formation (20–23).

Recent evidence suggests that the universal mechanism of epigenetic regulation, DNA methylation, is involved in bacterial persistence (24, 25). In particular, adenine methylation in GATC sequences by the DNA-(adenine N6)-methyltransferase (Dam) has been well studied as a major type of DNA modification in E. coli, Salmonella spp., *Yersinia* spp., and *Vibrio* spp. (26–28). Such reversible modifications do not alter the original DNA sequence but modulate epigenetic functions by regulating protein binding affinity (29, 30). However, the competition between transcription factors and methyltransferases (MTase) at specific motif sites can affect bacterial transcription (31, 32).

In the present study, we demonstrate that *dam*, coding a DNA-(adenine N6)-methyltransferase, is transcriptionally activated by various antibiotic pressures in A. baumannii. Moreover, the overexpression of *dam* from heterologous promoters leads to an increase in persister cell formation, along with a slow growth phenotype. In addition, Dam overexpression resulted in the transcriptional activation of several genes as possibly involved in persister cell formation, *recC*, *umuD*, *phoU*, and *glpD*. These results suggest that DNA methyltransferases could be important target proteins for the development of novel antibiotics to prevent the formation of persister cells.

## RESULTS

### Antibiotic susceptibility and persister cell formation.

The antibiotic susceptibility of the reference and clinical strains, and of their derivatives harboring plasmids are shown in Table S3. While the reference strain ATCC 19606 was resistant to aminoglycosides (amikacin and gentamicin), both clinical strains (C010 and C111) were susceptible to all the antibiotics used in this study, although they showed different MICs for some of them. The plasmid-harboring derivatives showed the same MICs as their parental strains, indicating that the cloned genes (*dam*, *recC*, *umuD*, *phoU*, and *glpD*) did not affect the antibiotic susceptibility of the bacterial host. For further investigation, we used the five antibiotics to which all strains showed susceptibility.

**FIG 1:**
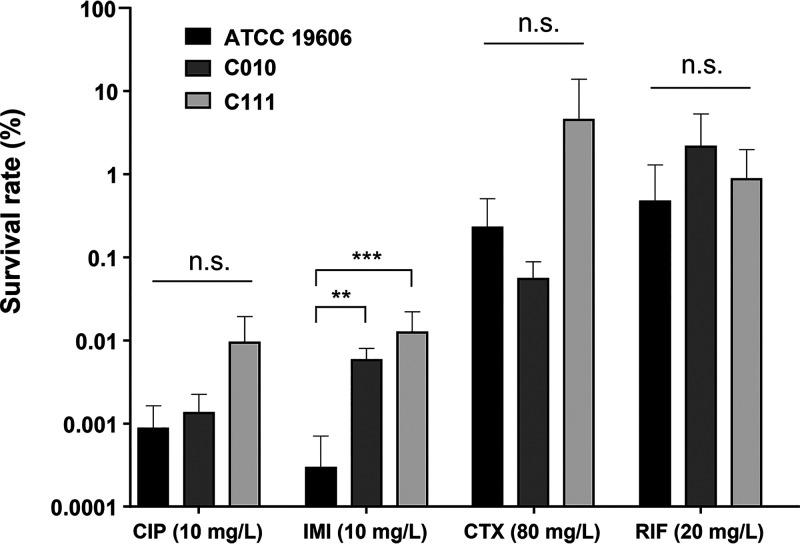
A. baumannii persister cell formation in the presence of various kinds of antibiotics. A. baumannii ATCC 19606 and clinical strains C010 and C111 were grown to exponential phase in LB medium. Persister cell formation rate was determined by culturing bacteria in the presence of 10 mg/L ciprofloxacin, 10 mg/L imipenem, 80 mg/L cefotaxime, or 20 mg/L rifampin for 6 h. Survival rate was calculated as described in Materials and Methods. Results are expressed as mean ± standard deviation from at least three independent experiments. CIP, ciprofloxacin; IMI, imipenem; CTX, cefotaxime; RIF, rifampin. **, *P < *0.01; ***, *P < *0.001; ns, not significantly different.

The persister cell formation rates of the three A. baumannii strains were evaluated using the five different antibiotics. Overall, between 0.0001% to 1% of parental cells survived when exposed to high concentrations of antibiotics, while preserving their antibiotic susceptibility (Fig. 1). Persister cell formation rates were relatively low in cultures containing ciprofloxacin and imipenem.

### Transcription levels of *dam* and persister cell-involved genes in bacteria exposed to antibiotics.

**FIG 2:**
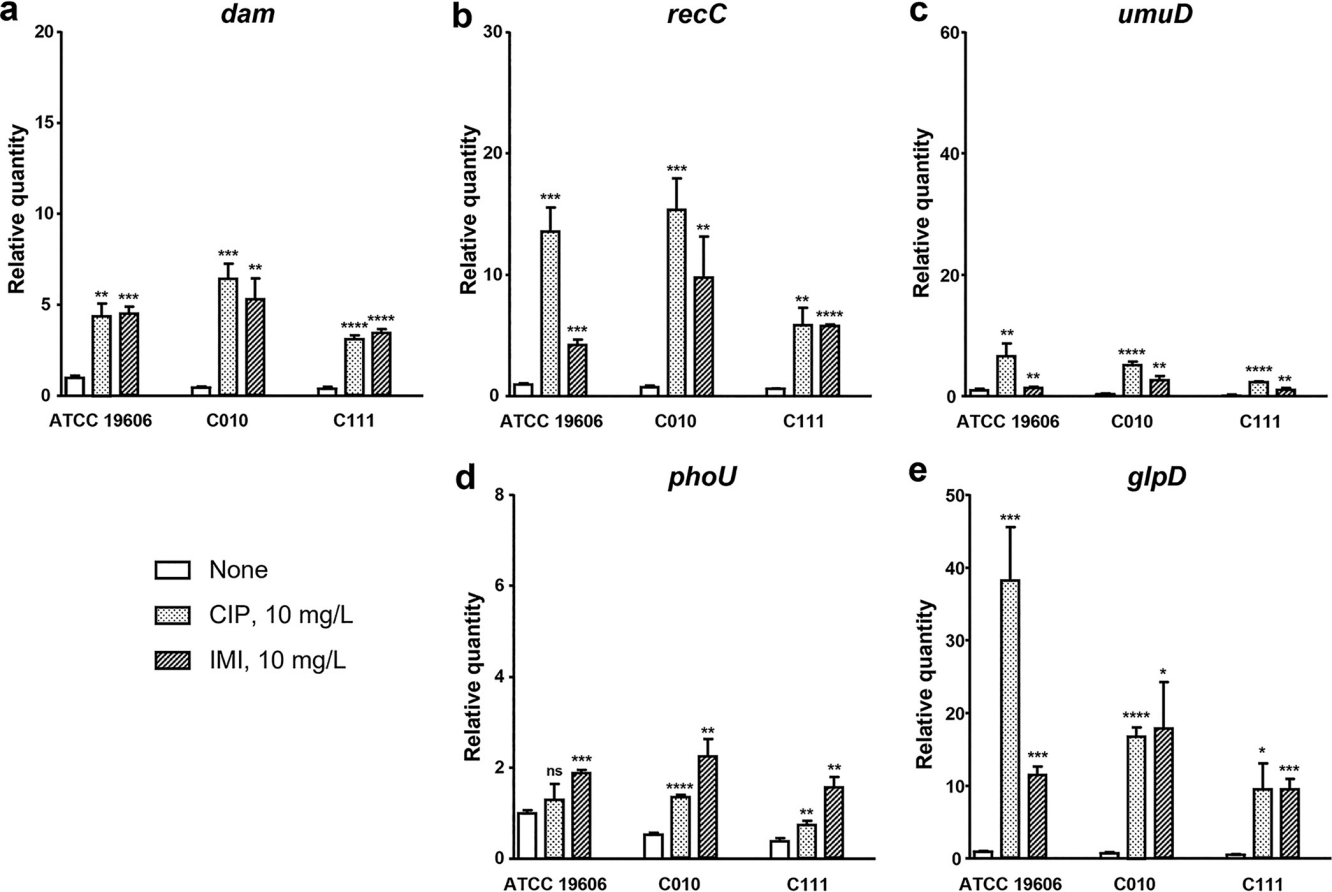
DNA methyltransferase (*dam*) and persister cell-involved genes are transcriptionally upregulated in the presence of antibiotics. A. baumannii strains were grown to exponential phase, exposed 10 mg/L ciprofloxacin (CIP) or 10 mg/L imipenem (IMI) for 6 h. The transcription levels of *dam* (a), *recC* (b), *umuD* (c) *phoU* (d), and *glpD* (e) were measured by qRT-PCR; the relative changes were calculated by the *ΔΔ*C_T_ method. Results are expressed as the mean ± the standard deviation from at least three independent experiments. *, *P < *0.05; **, *P < *0.01; ***, *P < *0.001; ****, *P < *0.0001; ns, not significantly different (relative to bacteria grown in antibiotic-free media; None).

We evaluated the transcriptional levels of *dam* and some persister cell-involved genes, previously reported in E. coli (19, 33–37), in strains exposed to ciprofloxacin and imipenem, *dam* expression increased significantly after 6 h of exposure to any of the three antibiotics (Fig. 2a). Cefotaxime and rifampin showed no significant difference. In addition, *recC*, *umuD*, *phoU*, and *glpD* transcription was activated when the strains were treated with antibiotics (Fig. 2b–2e). On the contrary, *sucB*, *recA*, and *fis* showed only slight changes in transcriptional levels, most of which were not significant (data not shown).

**FIG 3:**
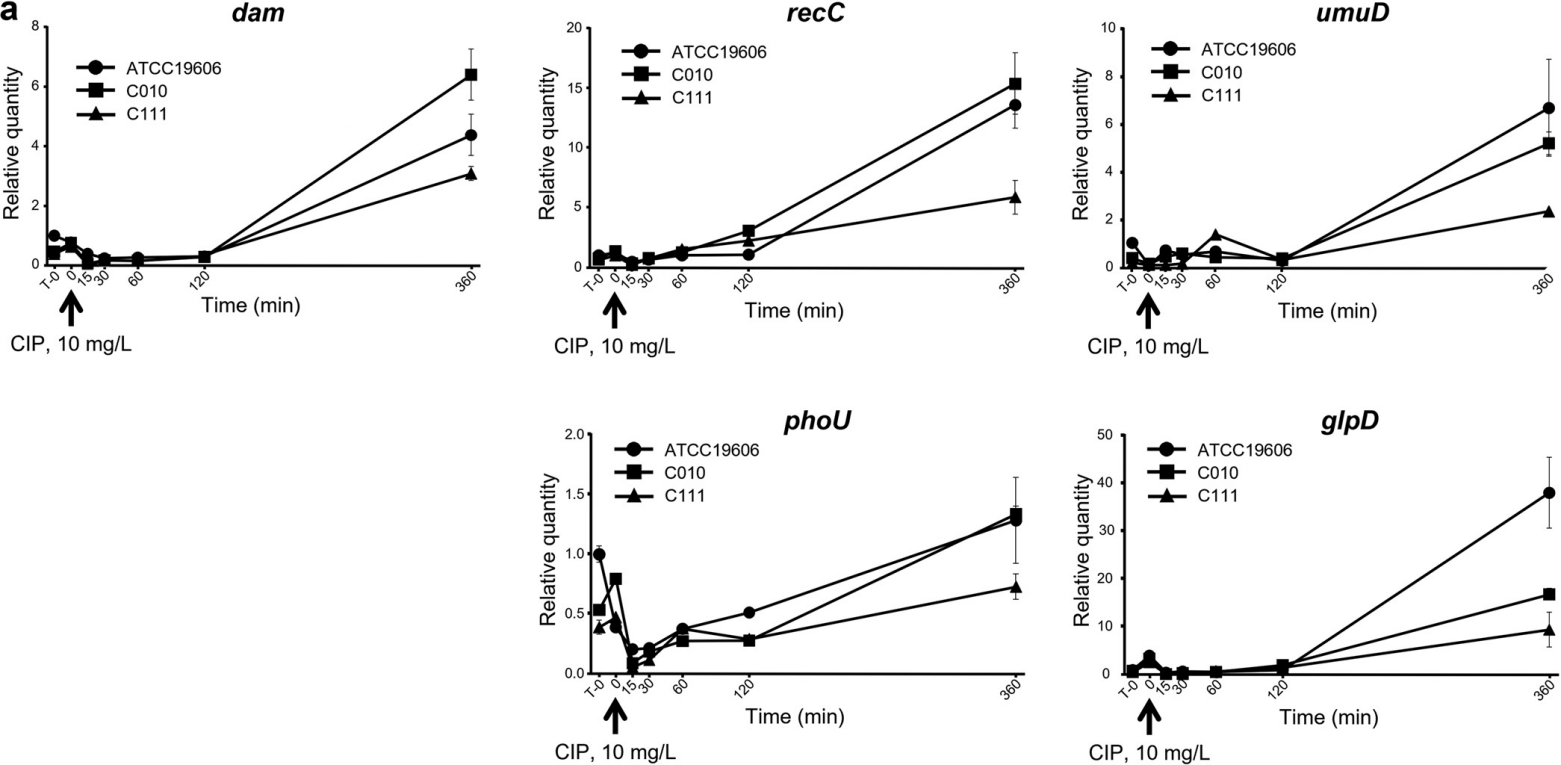
Transcription level changes in persister cell-involved genes over time. A. baumannii strains were grown to exponential phase, exposed to 10 mg/L ciprofloxacin for 6 h. Total mRNA was isolated at the following time points: T–0 (before antibiotic treatment), T + 0 (right after antibiotic treatment), and after 15, 30, 60, 120, and 360 min (T + 15, T + 30, T + 60, T + 120, and T + 360, respectively). The transcription levels of the persister cell-involved genes *dam*, *recC*, *umuD*, *phoU*, and *glpD* were determined by qRT-PCR. Results for T–0 and T + 360 are expressed as the mean ± the standard deviation of at least three independent experiments.

While the transcription of *dam*, *recC*, *umuD*, and *glpD* increased after 2 h of exposure to ciprofloxacin, the increase in *phoU* transcription began earlier (Fig. 3).

### Effect of Dam overexpression.

**FIG 4:**
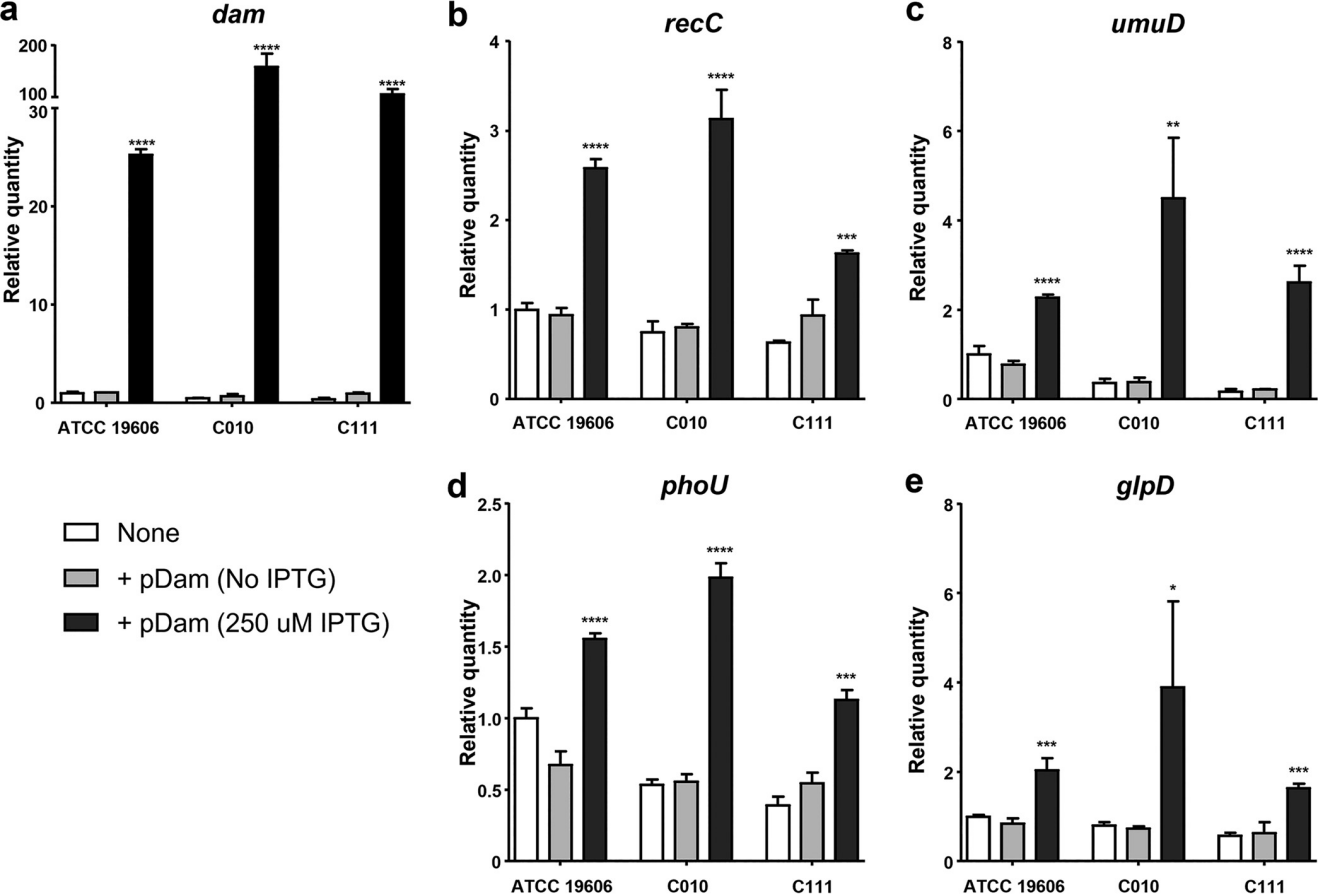
Overexpression of Dam contributes to the transcriptional activation of several persister cell-involved genes. A. baumannii strains harboring pDam were grown to exponential phase with or without 250 μM IPTG. The transcription levels of *dam* (a), *recC* (b), *umuD* (c) *phoU* (d), and *glpD* (e) were measured by qRT-PCR. Results are expressed as the mean ± the standard deviation from three independent experiments. *, *P < *0.05; **, *P < *0.01; ***, *P < *0.001; ****, *P < *0.0001; (relative to the uninduced strain harboring pDam; No IPTG).

To investigate the effect of Dam overexpression on persister cell formation and persister cell-involved gene expression, we constructed DNA-(adenine N6)-methyltransferase (Dam) overexpression strains using a gene overexpression system. The chromosomal gene RS07650(A1S_0222 in ATCC 17978), for which the Dam activity has been reported (38), was amplified in A. baumannii ATCC 19606 and cloned into a p*lac* plasmid. The pMAC origin of replication from ATCC 19606 was also cloned into the p*lac* plasmid vector to enable it to replicate in Acinetobacter spp. The selective antibiotic marker gene was changed, i.e., Kan^R^ substituted the ampicillin resistant gene (Amp^R^). The p*lac*-Dam was introduced into ATCC 19606, C010, and C111, generating ATCC 19606/pDam, C010/pDam, and C111/pDam, respectively. Dam was induced with 250 μM IPTG (Fig. 4a).

The transcription of all four persister cell-involved genes, *recC*, *umuD*, *phoU*, and *glpD*, increased significantly (1.5-fold to 5-fold) by IPTG induction in all three Dam-overexpressing strains (Fig. 4b to 4f), suggesting that increased levels of Dam may stimulate the expression of persister cell-involved genes. In addition, it is of note that the expression pattern of the four genes in Dam-overexpressing strains (+pDam with IPTG) (Fig. 4) was similar to that in bacteria exposed to high concentrations of antibiotics (Fig. 2). In contrast, transcription of *relA*, which contributes to the synthesis of the alarmone ppGpp, was not associated with Dam overexpression (Fig. S3). Although *relA* expression increased with Dam overexpression in C010/pDam and C111/pDam, it was not significant. Moreover, in ATCC 19606/pDam, *relA* expression decreased.

### Persister cell formation by Dam overexpression.

**FIG 5:**
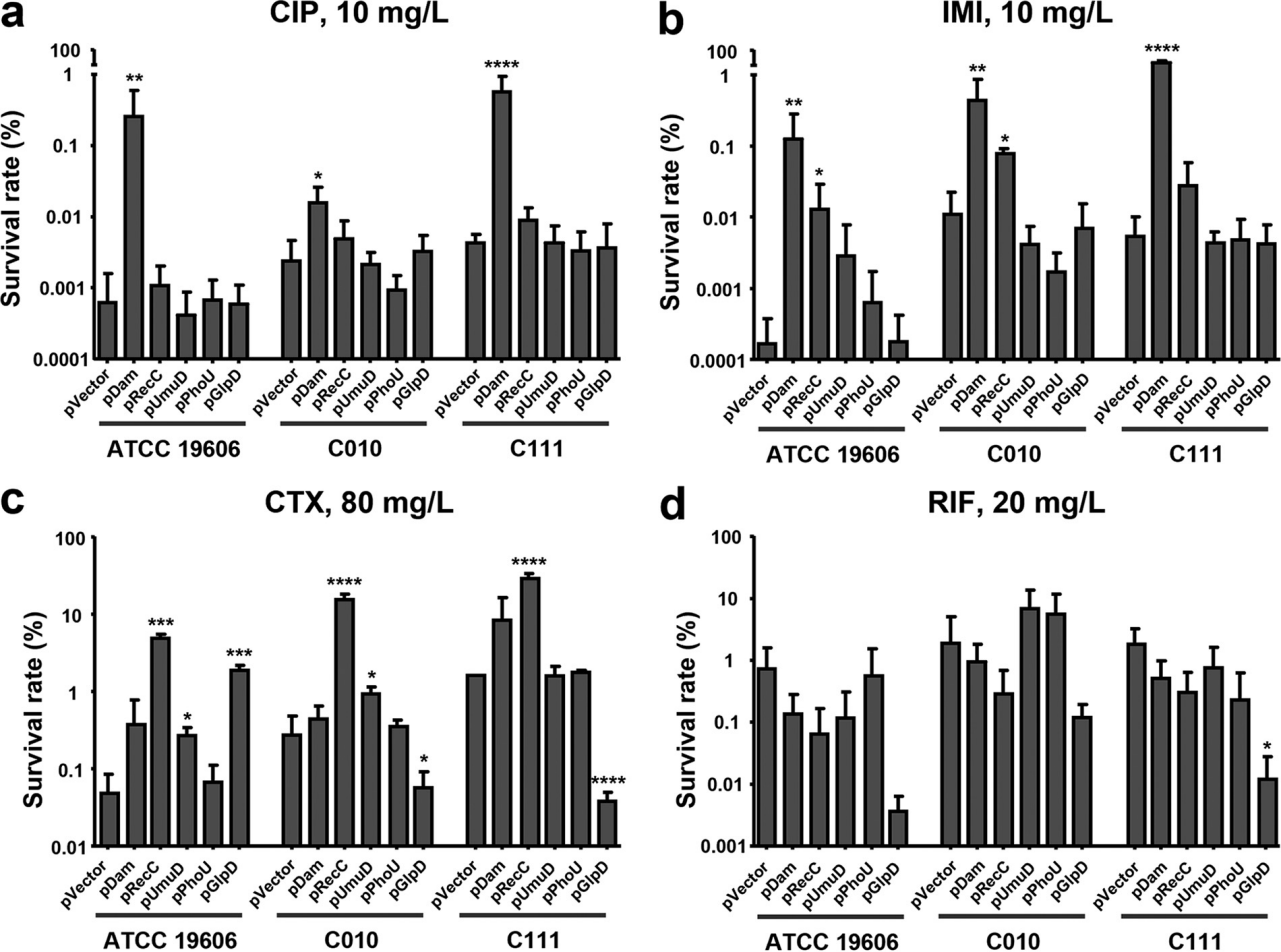
Persister cell formation rates in A. baumannii mutants overexpressing *dam*, *recC*, *umuD*, *phoU*, and *glpD*. Plasmids overexpressing either *dam* or any of the four persister cell-involved genes were constructed and introduced into strains ATCC 19606, C010, and C111. Plasmid encoded gene expression was induced with 250 μM IPTG. Persister cell formation assays were performed in the presence of 10 mg/L ciprofloxacin (a), 10 mg/L imipenem (b), 80 mg/L cefotaxime (c), or 20 mg/L rifampin (d). Results are expressed as the mean ± the standard deviation from at least three independent experiments. *, *P < *0.05; **, *P < *0.01; ***, *P < *0.001; ****, *P < *0.0001; (relative to the strain harboring each plasmid; pVector).

The rates of persister cell formation by several antibiotics were measured in dam-overexpressing strains. The persister cell formation rates increased significantly with Dam overexpression in the presence of ciprofloxacin, imipenem, and cefotaxime (Fig. 5a, 5b, and 4c). Moreover, persister cell formation in the presence of rifampin did not increase in any of the Dam-overexpressing strains (Fig. 5d).

### Transcription levels and persister cell formation in *Δdam*/pDam mutant.

We constructed *Δdam*/pDam mutant using the strain expressing the Dam from the plasmid-linked *lac* promoter as a recipient. However, persister cell formation slightly increased against ciprofloxacin, imipenem, and cefotaxime, compared with the wild type, even in the *Δdam*/pDam mutant with no IPTG (data not shown). The mRNA expression level of *dam* and other regulatory genes was determined in the *Δdam*/pDam mutant. Under the normal growth condition with no IPTG, the mRNA expression of *dam* was reduced 6-fold in the mutant strain, compared with wild type, and the expression of *recC*, *umuD*, *phoU*, and *glpD* were reduced about 2-fold, respectively (Fig. S4). After 6 h exposure of antibiotics with no IPTG, however, the absolute amount of mRNA expression of *dam* in *Δdam*/pDam mutant did not change compared to before exposure, but its relative expression was shown to increase due to much decrease of expression of *rpoB*, which was used a housekeeping gene to normalize the expression levels (Fig. S5). The mRNA expression of the other four genes was also shown to increase, although their absolute expression levels decreased (Fig. S5). These data also represent that the expression levels of *dam* and regulatory genes might be associated with persister cell formation rates.

### Persister cell formation by RecC overexpression.

We then explored which gene(s) were downstream regulated by Dam, leading to increased persister cell formation (Fig. 5). To construct RecC-, UmuD-, PhoU-, and GlpD-overexpressing strains, the *recC*, *umuD*, *phoU*, and *glpD* genes were amplified by PCR from the chromosome of A. baumannii ATCC 19606 and individually cloned into the p*lac* plasmid. The RecC-overexpressing strains showed elevated persister cell formation rates when cultured in the presence of imipenem and cefotaxime (Fig. 5b and 5c). However, persister cell formation by ciprofloxacin was not increased in RecC-overexpressing strains, in opposition to the results from Dam-overexpressing strains (Fig. 5a). UmuD overexpression was associated with an increase in persister cell formation in cefotaxime-containing media only (Fig. 5c). PhoU and GlpD overexpression showed no significant increase in persister cell formation following treatment with most antibiotics. Thus, the overexpression of the recombinational repair-associated protein RecC increases persister cell formation, acting together with or downstream of Dam.

### Effect of overexpressed genes on bacterial growth.

**FIG 6:**
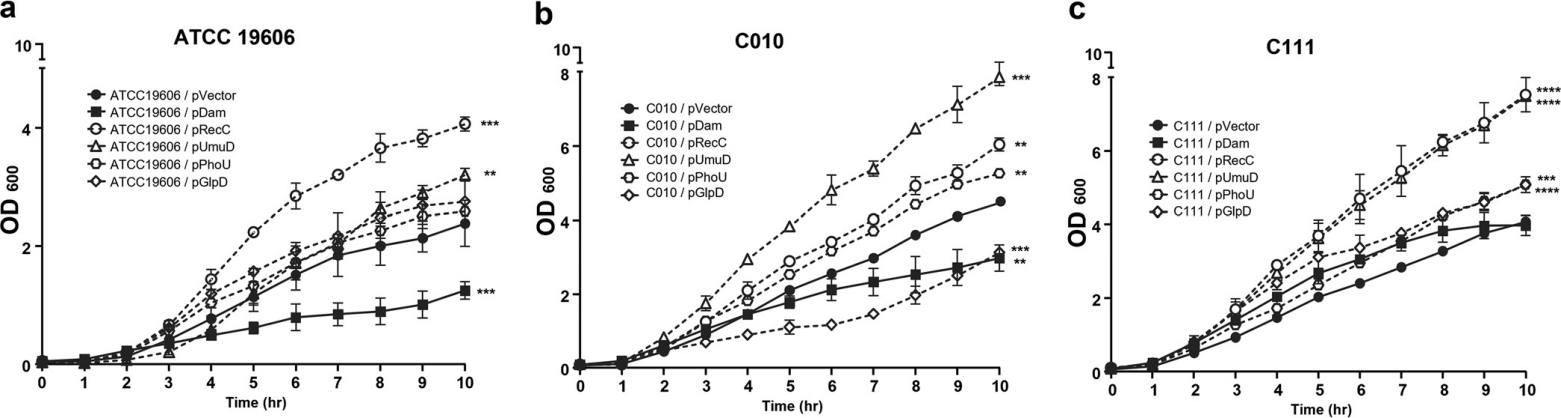
Growth of A. baumannii mutants overexpressing *dam*, *recC*, *umuD*, *phoU*, and *glpD*. Bacterial growth curves of A. baumannii ATCC 19606 (a) and the clinical strains C010 and C111 (b and c, respectively) harboring different plasmids and induced with 250 μM IPTG. Bacterial growth curves were initiated at 1:100 inoculation in 20 mL LB medium, the optical density was measured at λ = 600 (OD_600_) every single hour. Results are expressed as the mean ± the standard deviation from three independent experiments. **, *P < *0.01; ***, *P < *0.001; ****, *P < *0.0001; (relative to the strains with empty vectors).

We examined the growth of the A. baumannii strains and found that the Dam-overexpressing strains derived from ATCC 19606 and C010 showed lower growth rates than those bacteria carrying an empty vector (Fig. 6a and 6b) and the C111/pDam mutant (Fig. 6c). In contrast, RecC and UmuD overexpression significantly accelerated bacterial growth in all three strains (Fig. 6). Additionally, strains overexpressing PhoU and GlpD exhibited higher growth rates than the strains with no overexpression, but statistical significance was only observed in some of them.

## DISCUSSION

A. baumannii forms persister cells against various kinds of antibiotics and external environmental stresses, as shown here and in several previous studies (8, 39). The formation of persister cells has been suggested as one of the causes of antibiotic treatment failure (40). To date, several mechanisms of persister cell formation have been suggested (10, 41), and some global regulators have also been proposed (42). However, there are still many aspects to be elucidated regarding the formation and regulation of persister cells. In the present study, we aimed to investigate whether epigenetic regulation by DNA methylation affect the formation of persister cells in A. baumannii. As adenine methylation in GATC sequences by Dam has been well studied as a major type of epigenetic regulation in bacteria (43), we constructed A. baumannii strains overexpressing Dam.

The principal finding of this study was that Dam overexpression increased persister cell formation rates. Recently, DNA adenine methylation was reported to be involved in persister cell formation in uropathogenic E. coli (37). In that study, *dam^−^* mutants showed significant defects in persister cell formation. The results of our study showed that all A. baumannii strains overexpressing Dam exhibited increased survival rates against ciprofloxacin and imipenem with no change in antibiotic susceptibility, that is they showed elevated persister cell formation frequencies. Consistent with a previous study (37), our data suggest that persister cell formation is regulated epigenetically through DNA methylation. However, the increase in persister cell formation rate mediated by Dam overexpression was only observed for some of the antibiotics tested. Persister cell formation against fluoroquinolones and carbapenems increased in the strains with Dam overexpression, but this increase was not observed with other antibiotics. This confirms that the mechanisms of persister cell formation differ depending on the antibiotic (8). It is speculated that DNA methylation may modulate persister cell formation in A. baumannii, but this mechanism is only used in the presence of selected antibiotics. Additionally, the methylation changes by Dam overexpression, which would be determined by methylation sequencing such as SMRT, were not measured in this study. Whether methylation at any site is actually associated with persister cell formation should be investigated in further study, in addition to transcriptomic change associated with methylation.

Many regulatory genes have been reported to be involved in persister cell formation. *recC* and *umuD* gene products, which are essential in the SOS response of DNA repair systems, are known to induce persistence in E. coli (35, 36). *phoU* gene product, a global negative regulator of phosphate metabolism, has been reported to be involved in persister switching (19). The deletion of the energy metabolism-associated gene *glpD* has also been reported to reduce persister cell formation in E. coli (34). We observed that all these genes were transcriptionally upregulated by antibiotic stress at high concentrations, along with *dam*. While *dam*, *recC*, *umuD*, and *glpD* were overexpressed late after ciprofloxacin treatment, the expression of *phoU* began earlier. Our results suggest that these genes may be differentially regulated depending on the type of antibiotic and time elapsed since the beginning of antibiotic stimulation.

The transcriptional activation of these persister cell-involved genes might be mediated by Dam overexpression. This indicates that many mechanisms in bacterial physiology may be regulated epigenetically (24, 30). However, only RecC overexpression increased the persister cell formation rate against ciprofloxacin and imipenem in all three strains. Therefore, DNA methylation may affect persister cell formation in A. baumannii through the regulation of RecC.

However, the effect on bacterial growth rate was different between Dam- and Rec-overexpressing strains. While Dam overexpression decreased growth rates, RecC-overexpressing strains showed higher growth rates. Slow growth is known as a characteristic of persister cells in several bacterial species, including Staphylococcus aureus and Salmonella enterica (41, 44, 45). It has been shown that the growth rate decreases as bacteria enter the dormant state, forming persister cells (46). Thus, Dam overexpression may be associated with the initiation of dormancy. In contrast, the increased growth rate in strains with RecC overexpression may be explained by accelerated resuscitation (12). Persister cells should possess a means of resuscitation, since dormancy is a prevalent phenomenon in bacteria (11). Thus, both Dam and RecC contribute to the formation of persister cells in A. baumannii, and they seem to function in different states. Further studies on the process of persister cell formation and its regulation are required.

### Conclusions.

In this study, we showed that DNA methyltransferase levels affects persister cell formation in A. baumannii. Transcriptional activation of several persister cell-involved genes was shown to be mediated by the upregulation of the DNA methyltransferase. Of these, RecC was associated with an increase in persister cell formation mediated by Dam overexpression. In addition, DNA methyltransferase and RecC appear to act in different states of persister cell formation. Our results suggest that DNA methyltransferase can be an important target for the development of novel antibiotics to prevent the development of persister cells, which would contribute to retard the emergence of resistance.

## MATERIALS AND METHODS

### Bacterial strains, plasmids, and growth conditions.

The bacterial reference strain (ATCC 19606), clinical strains (C010 and C111), and plasmids used in this study are listed in Table S1. The clinical A. baumannii strains were collected from the Chonnam National University Hospital in 2003 and 2005. A. baumannii strains were grown at 37°C in Luria-Bertani (LB) medium or Mueller-Hinton (MH) broth. Bacteria were grown in Erlenmeyer flasks with vigorous shaking. Kanamycin (50 mg/L) was used to maintain plasmid-harboring strains. Isopropyl-β-d-thiogalactopyranoside (IPTG) was used at several concentrations to induce the expression of genes cloned under the control of the p*lac*.

### Plasmid construction.

We constructed plasmids p*lac*-Dam, p*lac*-RecC, p*lac*-UmuD, p*lac*-PhoU, and p*lac*-GlpD expressing the *dam*, *recC*, *umuD*, *phoU*, and *glpD* genes, respectively, under the control of the *lac* promoter. The genes were amplified from the chromosomal DNA of A. baumannii ATCC 19606 using the primer pairs EX-dam-F/EX-dam-R, EX-recC-F/EX-recC-R, EX-umuD-F/EX-umuD-R, EX-phoU-F/EX-phoU-R, and EX-glpD-F/EX-glpD-R, respectively (Table S2). The PCR products were purified and introduced between the BamHI and PstI restriction sites of the plasmid vector pUHE21-2*lacI*^q^ (47). The antibiotic resistance gene *bla* in pUHE21-2*lacI*^q^ was inactivated by restriction enzyme digestion with XmnI, the kanamycin resistance gene Kan^R^, which was obtained by PCR amplification from the plasmid pKD4 (48) with the primer pair EX-Km-F/EX-Km-R, was introduced. Since the pBR322 origin in pUHE21-2*lacI*^q^ cannot replicate in A. baumannii, the oriMAC origin was introduced in the vector at the XbaI restriction site; it was obtained by PCR amplification with the primer pair EX-oriMAC-F/EX-oriMAC-R using A. baumannii ATCC 19606 plasmid DNA as the template. To disrupt the ampicillin resistance *bla* gene in a gentamicin-resistant suicide vector pCVD442-Gen^R^, the Cm^R^ gene was introduced at the XmnI restriction site and named pHK1033. The chloramphenicol resistance Cm^R^ gene was obtained by PCR amplification from the plasmid pKD3 with the primer pair EX-Cm-F/EX-Cm-R (48). Primer sequences are listed in Table S2.

### Construction of *Δdam*/pDam strain.

The *dam* gene was deleted from the chromosome of the strain expressing the Dam from the plasmid-linked *lac* promoter (ATCC 19606 p-Dam). The 600 bp upstream (fragment A) and 600 bp downstream (fragment B) fragments of *dam* gene from the chromosomal DNA of A. baumannii ATCC 19606 were amplified using a primer pair DE-dam-A-F/DE-dam-A-R and DE-dam-B-F/DE-dam-B-R, respectively. Then, two DNA fragments were assembled by overlap-PCR. The resulting overlap-PCR products with c.a. 1,200 bp were integrated into the pHK1033 between SmaI and SphI restriction sites, resulting in a new plasmid named pHK263. An E. coli SM10 λ *pir^+^* strain containing the pHK263 was used as conjugal donor for ATCC 19606 pDam. Transconjugants were isolated onto LB agar plates containing ampicillin (150 mg/L) and gentamicin (50 mg/L) in order to eliminate the donor strain and to select the merodiploid strain. Plasmids with the antibiotic resistance cassette were eliminated from the bacterial strain, and antibiotic resistance cassette in the chromosome was excised by method or second crossover (49). Deletion of the *dam* gene was verified by PCR using primer pairs dam-check-F/dam-check-R and dam-F/dam-R, and nucleotide sequencing (Fig. S2). The sequences of primers used are listed in Table S2.

### Antimicrobial susceptibility testing.

Antimicrobial susceptibility testing was performed by broth microdilution method according to the Clinical and Laboratory Standards Institute guidelines (50). The MICs of eight antibiotics, including ciprofloxacin, imipenem, cefotaxime, rifampin, amikacin, gentamicin, and cefepime were determined. E. coli ATCC 25922 and P. aeruginosa ATCC 27853 were used as control strains.

### Persister cell formation assay.

Bacteria were inoculated 1:100 in 20 mL of LB medium with or without 50 mg/L of kanamycin and 250 μM IPTG and grown to an OD_600_ of 0.5. Each 2 mL of bacterial culture was removed to a 14 mL round bottom tube and then 5× or more MIC was added of either ciprofloxacin (10 mg/L), imipenem (10 mg/L), cefotaxime (80 mg/L), and rifampin (20 mg/L). Bacteria were then incubated at 37°C with vigorous shaking for an additional 6 h. After incubation, 1 mL bacterial culture samples were removed and washed twice with the same volume of phosphate buffer saline (PBS). Bacteria were diluted 10-fold by the serial dilution method on 96-well plates, each 10 μL was spotted on a pure LB agar plate, and the total number of CFU (CFU) was calculated after incubation at 37°C for 16 h. The persister cell formation rate was calculated by normalization with T-0 (number of CFU from samples taken immediately before antibiotic exposure).

Based on *in vitro* time-killing curve, we confirmed that exposure to antibiotics for 6 h is enough to measure the persister rate (Fig. S2). To ensure that they are true persister and did not arise as a consequence of spontaneous resistance mutations, two colonies obtained from the persister formation assay were inoculated in 3 mL pure LB broth and incubated at 37°C with shaking overnight. The next day, MICs were determined for bacterial cultures and compared with those of the parental strains. The colonies were designated persister cells when the MIC value did not change.

### RNA isolation and quantitative real-time RT-PCR (qRT-PCR).

Bacteria were grown in LB medium with or without 50 mg/L of kanamycin and 250 μM IPTG to an OD_600_ of 0.5. The culture (0.5 mL) was removed and mixed with 1 mL RNAprotect bacterial reagent (Qiagen, Hilden, Germany), and RNA was isolated using a RNeasy minikit (Qiagen). After RNase-free DNase (Invitrogen) treatment, cDNA was synthesized from 1 μg of template RNA using Omniscript Reverse Transcription reagents (Qiagen) and random primers (Invitrogen). The amount of cDNA was quantified by qRT-PCR using the TB Green Premix Ex Taq (TaKaRa) with a QuantStudio 6 Flex System (ThermoFisher Scientific). The *ΔΔ*C_T_ values of the target genes were calculated using the C_T_ values of the reference strains and the housekeeping gene *rpoB*. To detect the cDNA corresponding to *dam*, *recC*, *umuD*, *phoU*, *glpD*, *sucB*, *recA*, and *fis*, and *rpoB* mRNAs, the primer pairs presented in Table S2 were used, respectively.

### Time course RNA isolation and qRT-PCR.

Bacteria were grown in LB medium to an OD_600_ of 0.5. Bacterial cultures (10 mL each) were then transferred to new Erlenmeyer flasks and incubated with either 10 mg/L ciprofloxacin for 6 h. During this incubation, bacterial mRNA was isolated at different time points (0, 15, 30, 60, 120, and 360 min), and qRT-PCR was performed as described above.

### Statistical analysis.

Statistical analyses were performed using GraphPad Prism version 8.3. The results were analyzed using the *unpaired t test*. The data are presented as the mean ± the standard deviation. A *P* value of <0.05 was considered statistically significant.
