# Supplementary material for: Overexpression of a DNA Methyltransferase Increases Persister Cell Formation in Acinetobacter baumannii
Source: Microbiol Spectr. 2022 Nov 23;10(6):e02655-22. doi: 10.1128/spectrum.02655-22 (PMC9769888; doi:10.1128/spectrum.02655-22)
Supplement: Supplemental file 1 — Tables S1 to S3 and Fig. S1 to S5. Download spectrum.02655-22-s0001.pdf, PDF file, 0.6 MB [file spectrum.02655-22-s0001.pdf]

Supplementary Table S1. Bacterial strains and plasmids used in this study

Strains or plasmids	Genotype or relevant characteristics	Reference or source
Strains or isolates		
ATCC 19606	*A. baumannii* reference strain	
19606-p	ATCC 19606 / p*lac* Kan^R oriMAC	This study
19606-pDam	ATCC 19606 / p*lac*-Dam Kan^R oriMAC	This study
19606-pRecC	ATCC 19606 / p*lac*-RecC Kan^R oriMAC	This study
19606-pUmuD	ATCC 19606 / p*lac*-UmuD Kan^R oriMAC	This study
19606-pPhoU	ATCC 19606 / p*lac*-PhoU Kan^R oriMAC	This study
19606-pGlpD	ATCC 19606 / p*lac*-GlpD Kan^R oriMAC	This study
C010	*A. baumannii* clinical isolate	Chung and Ko, 2019
C010-p	C010 / p*lac* Kan^R oriMAC	This study
C010-pDam	C010 / p*lac*-Dam Kan^R oriMAC	This study
C010-pRecC	C010 / p*lac*-RecC Kan^R oriMAC	This study
C010-pUmuD	C010 / p*lac*-UmuD Kan^R oriMAC	This study
C010-pPhoU	C010 / p*lac*-PhoU Kan^R oriMAC	This study
C010-pGlpD	C010 / p*lac*-GlpD Kan^R oriMAC	This study
C111	*A. baumannii* clinical isolate	Chung and Ko, 2019
C111-p	C111 / p*lac* Kan^R oriMAC	This study
C111-pDam	C111 / p*lac*-Dam Kan^R oriMAC	This study
C111-pRecC	C111 / p*lac*-RecC Kan^R oriMAC	This study
C111-pUmuD	C111 / p*lac*-UmuD Kan^R oriMAC	This study
C111-pPhoU	C111 / p*lac*-PhoU KanR oriMAC	This study
C111-pGlpD	C111 / p*lac*-GlpD Kan^R oriMAC	This study
Plasmids		
pUHE21-2*lacI*q	P_{lac} rep_{pMBI} Ap^R $lacI^q$	Soncini et al., 1995
pHK1021	pUHE21-2$lacI^q$ Kan^R	This study
pHK1024	pUHE21-2$lacI^q$ Kan^R oriMAC	This study
pHK247	pUHE21-*2*$lacI^q$ *dam*	This study
pHK248	pUHE21-*2*$lacI^q$ *dam* oriMAC	This study
pHK250	pUHE21-2$lacI^q$ *dam* Kan^R oriMAC	This study
pHK258	pUHE21-2$lacI^q$ *umuD* Kan^R oriMAC	This study
pHK259	pUHE21-2$lacI^q$ *dglpD* Kan^R oriMAC	This study
pHK260	pUHE21-2$lacI^q$ *phoU* Kan^R oriMAC	This study
pHK261	pUHE21-2$lacI^q$ *recC* Kan^R oriMAC	This study
pHK1033	pCVD442-Cm^R-$Gent^R$	This study
pHK263	pCVD442-Cm^R-$Gent^R$-del-dam	This study

Supplementary Table S2. Primers used in this study.

Primers	Sequences (5' to 3')
EX-dam-F	TGAGAGGATCCATGAATTCAGAGCCTTCGGTATA
EX-dam-R	CTTGGCTGCAGTTACCAAAGTGCGAGCTGTGTAC
EX-recC-F	TGAGAGGATCCATGGGTATCCATGTTATTCA
EX-recC-R	TCGACGGATCCTTACTCCGAATGTTGAAATTG
EX-umuD-F	TGAGAGGATCCATGCCAAAGAAGAAAGAATTCGAGC
EX-umuD-R	TCGACGGATCCTTATCTCATTCGTTTGAGGT
EX-phoU-F	TGAGAGGATCCTTGAGTCCGAGTAATCCGGT
EX-phoU-R	TCGACGGATCCTTACTTTTCGTGTACTTTTGC
EX-glpD-F	TGAGAGGATCCATGAAAGTACAACCTAATGA
EX-glpD-R	TCGACGGATCCTTATGCGGCATCATCTTTTAATCG
EX-Km-F	CCCGAAGAACGTTTTCAAGATCCCCTCACGCTGCCGCAAG
EX-Km-R	TCATTGGAAAACGTTCAGAGCGCTTTTGAAGCTGGGGTGG
EX-oriMAC-F	TGGTGTCTAGATTCATTGGGCTTTTAATTAT
EX-oriMAC-R	TCGACTCTAGAGTGTAAATCTCTGCCTAATC
EX-Cm-F	CCCGAAGAACGTTTTCGAATAGGAACTTCATTTAAATGGCG
EX-Cm-R	TCATTGGAAAACGTTCGGCGCGCCTACCTGTGACGGAAGAT
DE-dam-A-F	GAATTCCCGGGGGACGTACTGCAATGGCATC
DE-dam-A-R	ATTGCAGCGCTCATACTTTCTCTTGCGTAG
DE-dam-B-F	GAAAGTATGAGCGCTGCAATTTAAGCAGTG
DE-dam-B-R	GTACCGCATGCTGCTAAAACATCAACTTCAA
dam-check-F	GCTTGTTGCAGGTAAAGGTG
dam-check-R	CGGAACGTATGTCCGGTTTT
dam-F	GGCAAAGTGCTAGAGCCATGTTC
dam-R	CACATCGCCACCTACACTTTGTA
Q-Ac-dam-F	TCAGAGCCTTCGGTATACCACAA
Q-Ac-dam-R	TACGGGACAAGCTGATGGAAA
Q-Ac-recC-F	GCAGGGCGTACTGGCATCTA
Q-Ac-recC-R	ATGGCAGGACTTGGCACAAT
Q-Ac-umuD-F	TCAAAACCGATAACCAGACAGATTT
Q-Ac-umuD-R	CGTTCTGTCGCCAAAGGAAT
Q-Ac-phoU-F	GAAGATTTGCAAGATGTGAACACAA
Q-Ac-phoU-R	TCGCATCTGTATCAAGCAAAGAA
Q-Ac-glpD-F	AAGATGATTTAGCCAGCCATACCT
Q-Ac-glpD-R	GCTTCTCTGACCAGCCTGAATT
Q-Ac-sucB-F	TTAGGCTTCATGTCATTCTTCGTT
Q-Ac-sucB-R	CGCCATCAATTGAAGCATTTAC
Q-Ac-recA-F	TGGTACATGTGCCTTCATTGATG
Q-Ac-recA-R	GTCGGGTTGTGAAACAAGTAGGT
Q-Ac-fis-F	GATGTTGCTCTTCGCATCCA
Q-Ac-fis-R	ACCTGAGATGGTTGCTCACCTT
Q-Ac-relA-F	CTGAGTTGTCAGAGGAAACGACTGTA
Q-Ac-relA-R	ACGAACCCGATCCAACCAT
Q-Ac-rpoB-F	GCAAGATGGCAAATCACCAA
Q-Ac-rpoB-R	TTCTAAAGCAGCATTGCCAGAA

Supplementary Table S3. Antibiotic susceptibility profiles of *A. baumannii* strains or isolates

Strains or isolates	MICs (mg/L)[a]							
	CIP	IMP	TET	CTX	CPM	AMK	GEN	RIF
ATCC 19606	2	0.25	1	16	8	64	16	1
19606-p	2	0.25	1	16	8	64	16	1
19606-pDam	2	0.25	1	16	8	64	16	1
19606-pRecC	2	0.25	1	16	8	64	16	1
19606-pUmuD	2	0.25	1	16	8	64	16	1
19606-pPhoU	2	0.25	1	16	8	64	16	1
19606-pGlpD	2	0.25	1	16	8	64	16	1
C010	0.125	0.25	0.25	2	0.5	4	1	0.5
C010-p	0.125	0.25	0.25	2	0.5	4	1	0.5
C010-pDam	0.125	0.25	0.25	2	0.5	4	1	0.5
C010-pRecC	0.125	0.25	0.25	2	0.5	4	1	0.5
C010-pUmuD	0.125	0.25	0.25	2	0.5	4	1	0.5
C010-pPhoU	0.125	0.25	0.25	2	0.5	4	1	0.5
C010-pGlpD	0.125	0.25	0.25	2	0.5	4	1	0.5
C111	0.25	0.25	1	16	4	8	1	4
C111-p	0.25	0.25	1	16	4	8	1	4
C111-pDam	0.25	0.25	1	16	4	8	1	4
C111-pRecC	0.25	0.25	1	16	4	8	1	4
C111-pUmuD	0.25	0.25	1	16	4	8	1	4
C111-pPhoU	0.25	0.25	1	16	4	8	1	4
C111-pGlpD	0.25	0.25	1	16	4	8	1	4

[a] MIC, minimum inhibitory concentration; CIP, ciprofloxacin; IMI, imipenem; TET, tetracycline; CTX, cefotaxime; CPM, cefepime; AMK, amikacin; GEN, gentamicin; RIF, rifampin.

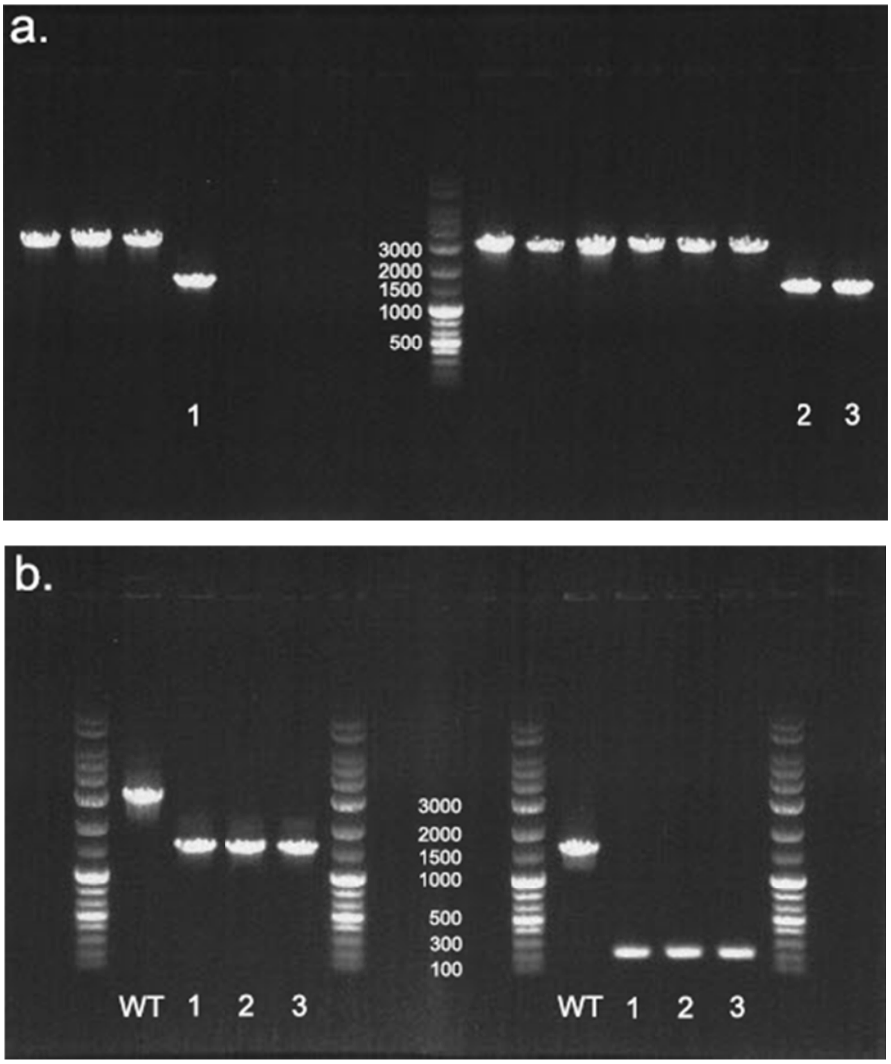

Supplementary Figure S1. Construction of chromosomal *dam* deletion mutant in *A. baumannii* ATCC 19606 strain harboring pDam plasmid. After the second crossover, colonies were isolated onto LB agar plates with 10% sucrose and without NaCl. Then, deletion of chromosomal *dam* gene was confirmed by PCR using primer pairs dam-check-F/dam-check-R (a and b-left, WT 2,792 bp; *Δdam*, 1,547 bp) and dam-F/dam-R (b-right, WT 1,531 bp; *Δdam*, 242 bp). 1, 2, and 3 represent *Δdam* mutants.

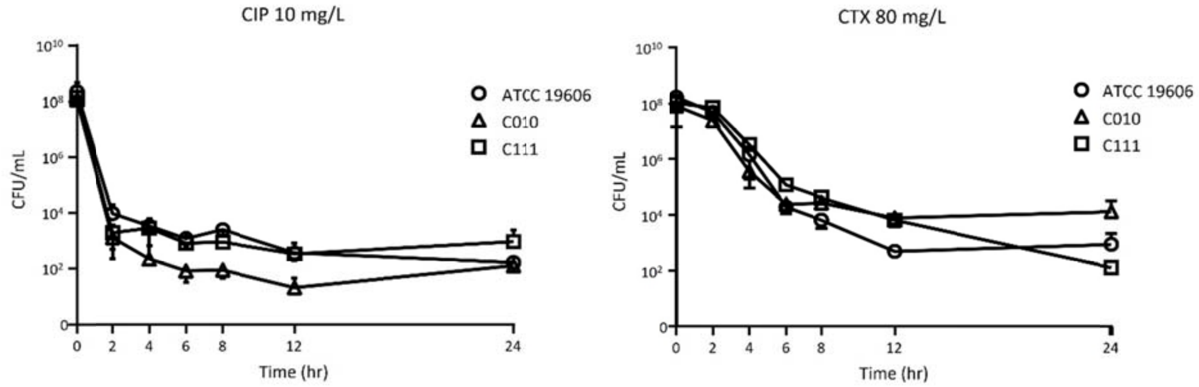

Supplementary Figure S2. In vitro time-killing assay for three *A. baumannii* strains using two antibiotics, ciprofloxacin (10 mg/L) and cefotaxime (80 mg/L). After exposure to antibiotics for 6 h, plateau of biphasic killing curve was shown.

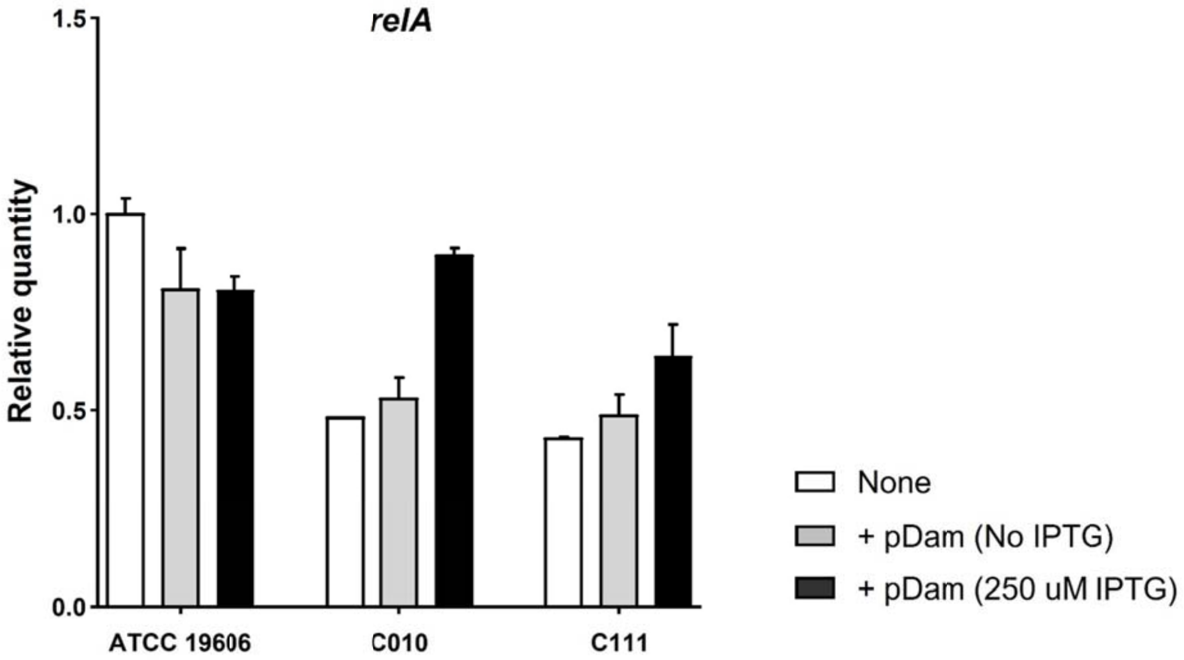

Supplementary Figure S3. Dam overexpression does not affect *relA* transcription levels. *A. baumannii* strains with or without plasmid p*lac*-Dam were grown to exponential phase in the presence or absence of IPTG 250 µM. The transcription levels of *relA* were determined by qRT-PCR in each strain. Results are expressed as the mean ± the standard deviation from three independent experiments.

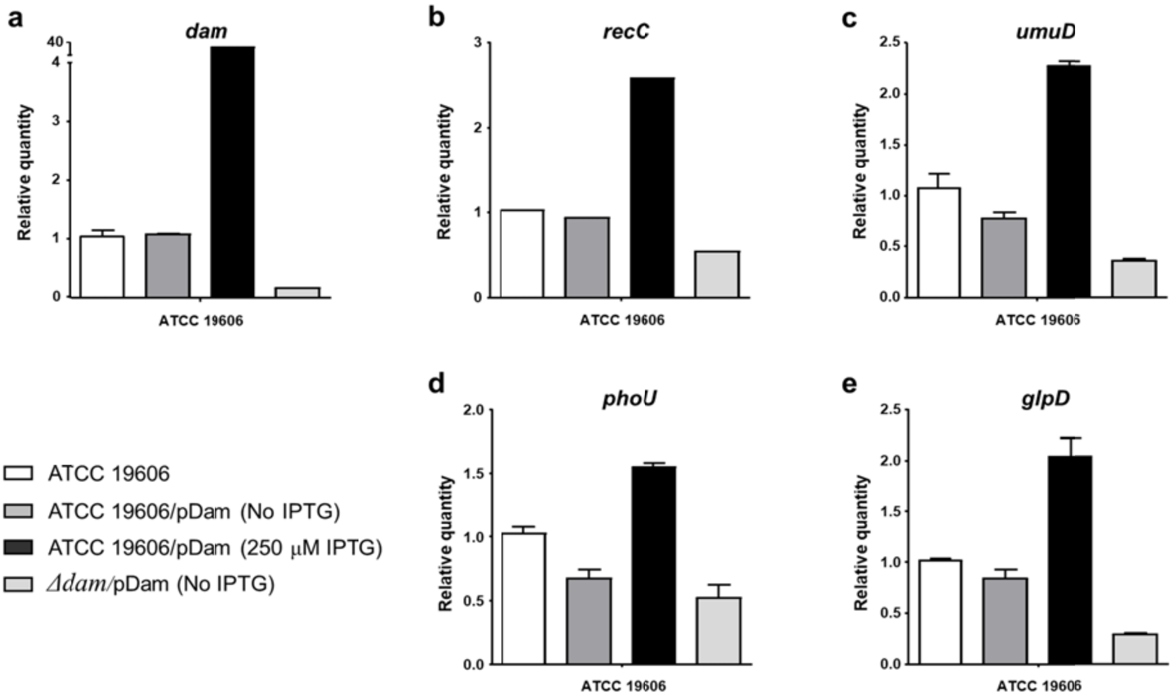

Supplementary Figure S4. Deletion of chromosomal *dam* contributes to the transcriptional inactivation of several persister cell-involved genes. *A. baumannii* ATCC 19606 wild-type strain, the strain harboring pDam plasmid (ATCC 19606/pDam), and *Δdam*/pDam mutant were grown to exponential phase with or without IPTG. The transcription levels of *dam* (a), *recC* (b), *umuD* (c) *phoU* (d), and *glpD* (e) were measured by qRT-PCR. Results are expressed as the mean ± the standard deviation from three independent experiments.

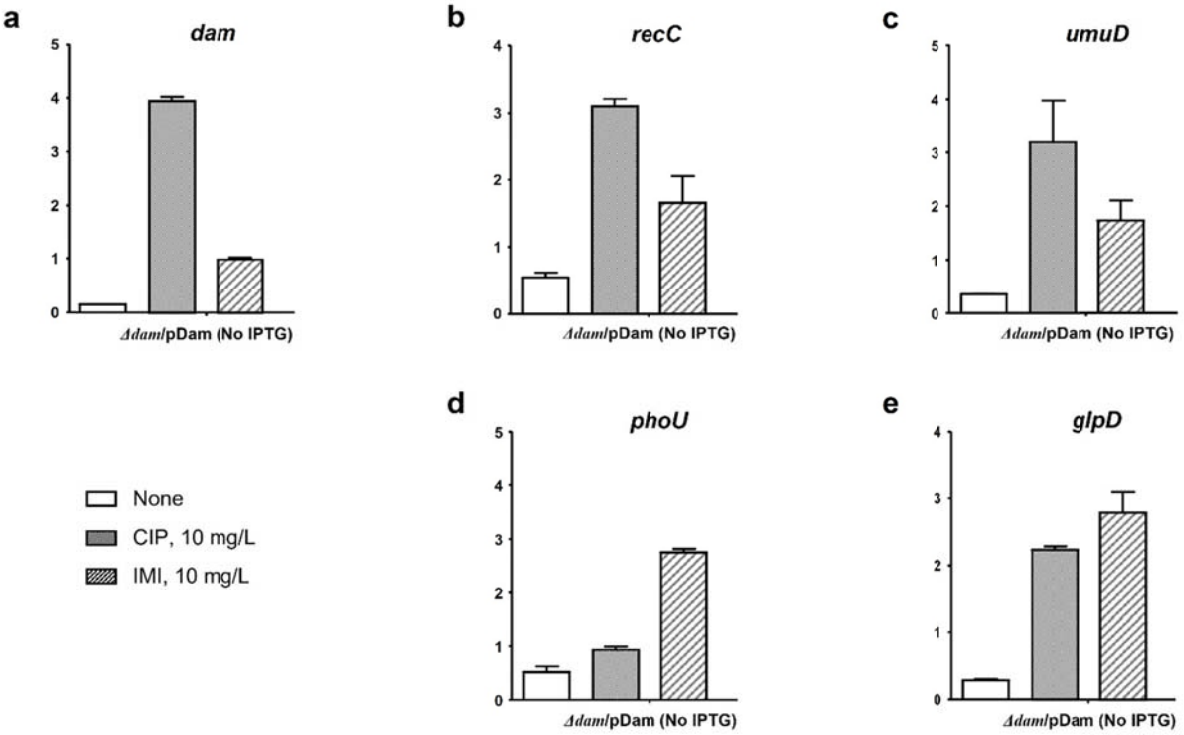

Supplementary Figure S5. DNA methyltransferase (*dam*) from the plasmid-linked *lac* promoter and persister cell-involved genes are transcriptionally upregulated in the presence of antibiotics. *Δdam*/pDam mutants were grown to exponential phase with no IPTG, under exposure of antibiotics (10 mg/L ciprofloxacin (CIP), or 10 mg/L imipenem (IMI)) for 6 h. The transcription levels of *dam* (a), *recC* (b), *umuD* (c) *phoU* (d), and *glpD* (e) were measured by qRT-PCR; the relative changes were calculated by the $\Delta\Delta C_T$ method. Results are expressed as the mean ± the standard deviation from at least three independent experiments.

References

Chung ES, Ko KS. Eradication of persister cells through antibiotic combination of colistin and amikacin in *Acinetobacter baumannii*. *J Antimicrob Chemother.* 2019; 74: 1277–83.

Soncini FC, Vescovi EG, Groisman EA. Transcriptional autoregulation of the *Salmonella* Typhimurium *phoPQ* operon. *J Bacteriol.* 1995; 177:4364–4371.
